
JOURNAL INFORMATION
==============================
NLM Title Abbreviation: Atlanta Med Surg J (1884)
NLM Journal ID: 101251623

Atlanta Medical and Surgical Journal (1884)

Publisher: Biomedical Journal Digitization Project

ARTICLE INFORMATION
==============================
PMCID: PMC9201866
Article version: 1
Subjects: Cover
Print publication date: 1899 Feb
Volume: 15
Volume Series: New Ser
Issue: 12
License: This work is free of known copyright restrictions. For more information, please see PMC Back Issue Digitization.
License URL: https://creativecommons.org/publicdomain/mark/1.0/

==============================

PHILLIPS' PHOSPHO-MURIATE OF QUININE, COMP.
(the Soluble Phosphates, with Muriate of Quinine, Iron and Strychnia)
IN DEFICIENCY OF THE PHOSPHATES, LACK OF NERVE TONE, MALARIAL MANIFESTATIONS, CONVALESCENCE FROM EXANTHEMATA, ETC.—WILL NEVER DISAPPOINT.
BEWARE OF THE MANY IMITATIONS. PRESCRIBE "PHILLIPS'."
THE CHAS. H. PHILLIPS CHEMICAL CO., 77 Pine Street, NEW YORK.
Please mention this Journal.

LIBRARY Indexed S. G. O.

ESTABLISHED 1855. SUBSCRIPTION $1.00.

LIBRARY
SURGEON GENERAL'S OFFICE
FEB.-8--1899

### ATLANTA MEDICAL AND SURGICAL JOURNAL.

Organ of
Atlanta Society of Medicine
and
State Examining Board.

VOLUME XV
NEW SERIES. ATLANTA, GA., FEBRUARY, 1899. No. 12.

"FIKULAX" (Ficus Chocolatis Laxans) is presented as a laxative which causes a gentle, alvine evacuation without irritation. Most valuable in habitual constipation.
FIKULAX is delightful in taste and attractive in appearance, important attributes in a medicament to be administered to women and children.

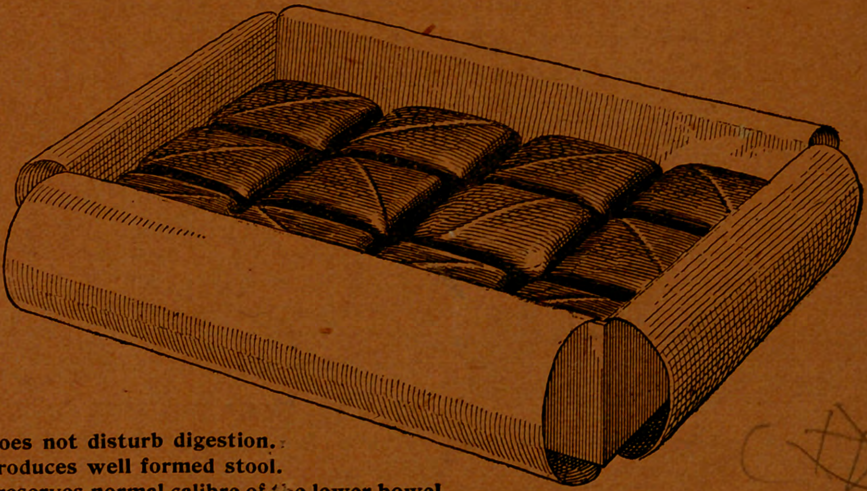

Does not disturb digestion.
Produces well formed stool.
Preserves normal calibre of the lower bowel.
CHAS. ROOME PARMELE CO., 36 PLATT STREET, NEW YORK.
Please mention this Journal.
